# Dr. Jian Zhou: The great inventor of cervical cancer vaccine

**DOI:** 10.1007/s13238-016-0358-2

**Published:** 2017-01-20

**Authors:** Kong-Nan Zhao, Lifang Zhang, Jia Qu

**Affiliations:** 1 Institute of Molecular Virology and Immunology, Wenzhou Medical University, Wenzhou, 325035 China; 2 Centre for Clinical Research, Translational Research Institute, The University of Queensland School of Medicine, 37 Kent Street, Woolloongabba, Brisbane, QLD 4102 Australia; 3 Laboratory of Neurovascular Biology, School of Ophthalmology and Optometry and the Eye Hospital of Wenzhou Medical University, Wenzhou, 325003 China; 4 The State Key Laboratory Cultivation Base and Key Laboratory of Vision Science, Ministry of Health, Wenzhou, 325027 China

**Figure 1:**
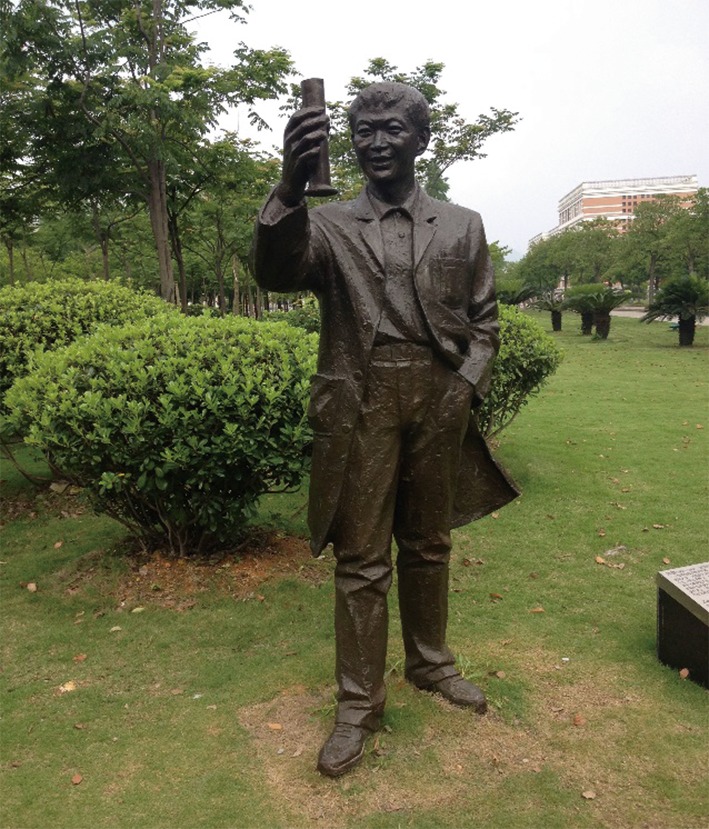
Statue photo of Dr. Jian Zhou on the campus of Wenzhou Medical University

In November 2005, scientists gathered at Times Square in New York City announced the exciting news of the successful development of the world’s first cervical cancer vaccine. The cervical cancer vaccine, which is able to protect unexposed women against the infection with four HPV strains responsible for 70% of cervical cancers, was approved for human use by the US Federal Drug Administration (FDA) and a number of other countries. The Times magazine reported the development of the cervical cancer vaccine as one of the world’s top ten scientific and technological achievements of 2006. In August 2006, the first dose of the vaccine was administered by Professor Ian Frazer in the presence of Anna Bligh, the Premier of Queensland. Anna Bligh said “Today we are making medical history. It is a great moment for science in this country.” Professor Frazer said that this historic breakthrough could not have been achieved without the contribution of Dr. Jian Zhou (Fig. 1). Sadly, Dr. Zhou, the great inventor of the vaccine, passed away in 1999 and was unable to see the benefits brought by the vaccine.

**Figure 2:**
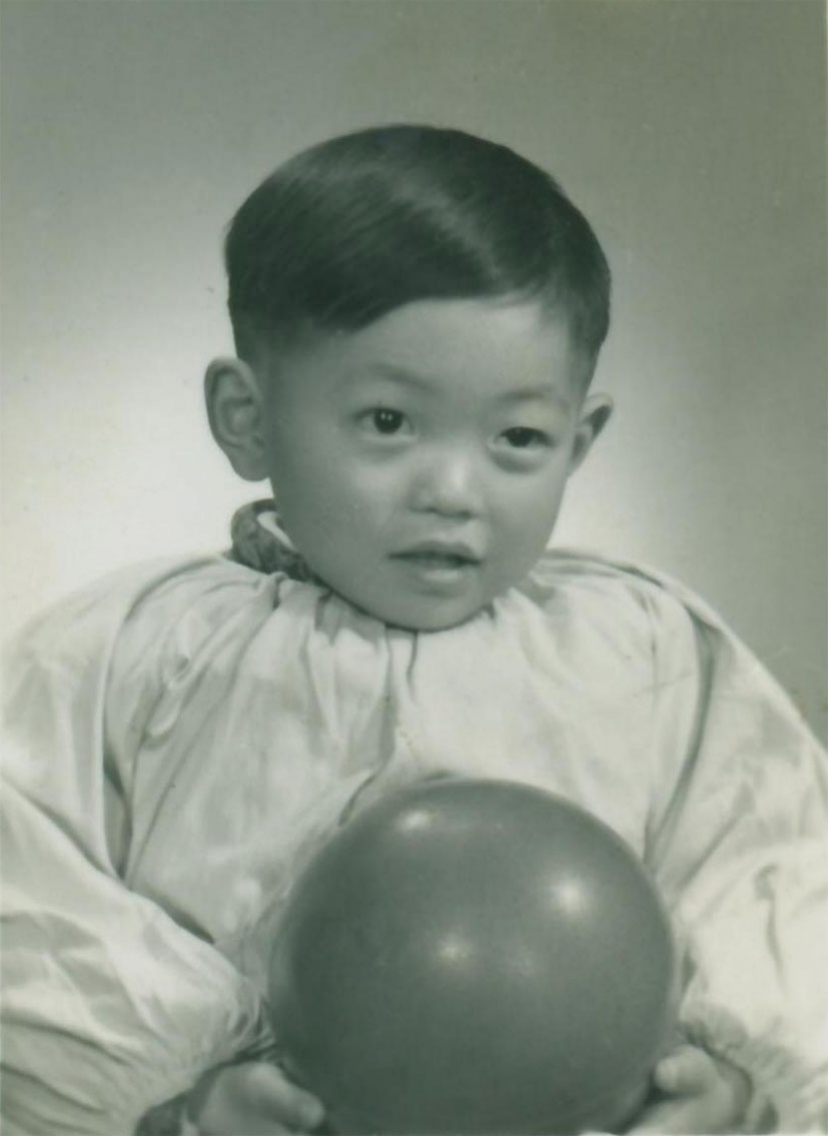
Dr. Jian Zhou’s childhood photo

Dr. Zhou was born in 1957 in Hangzhou, Zhejiang Province, China. At the time of his birth, his father was already 50 years old and the birth of a boy brought the family hope and joy. As a child, Dr. Zhou was active and playful and was often asked to leave his kindergarten classroom because he was busy in doing and playing by himself (Fig. 2). However, the punishment did not seem to affect the young Dr. Zhou and he was often seen having a good time playing by himself outside of the classroom. Even as a child, he seemed to have inexhaustible energy and this energy, an important factor that contributed to his later success, stayed with him all of his life.

Dr. Zhou spent his childhood in Hangzhou. After graduating from high school, he was sent to work in a rural village as a consequence of the “Cultural Revolution” in China. There was no school to attend. During his time working in the village, Dr. Zhou gained profound empathy for the hardships experienced by farmers. After working on farms for a year, he returned to Hangzhou as a temporary worker at the “June-One Knitting Factory”. There, he pulled carts and carried packages weighing more than 50 kilograms for a measly eighty cents a day. Although the work was extremely hard, Dr. Zhou never lost his spirit. Later, Dr. Zhou worked as a welding apprentice at a radio factory. Because of his youth and lanky build, a number of team leaders at the factory were reluctant to accept him as their apprentice. However, one team leader noticed that he was very smart, more thoughtful than others and had skilful hands—he was able to grasp the welding technology and produce high quality products quickly. This team leader took the young Dr. Zhou on and relied on him as a core team member until he left the factory to attend university. Dr. Zhou’s experiences of working on farms and in factories cultivated his ability to endure hardships and his dedication to the development of science.

**Figure 3:**
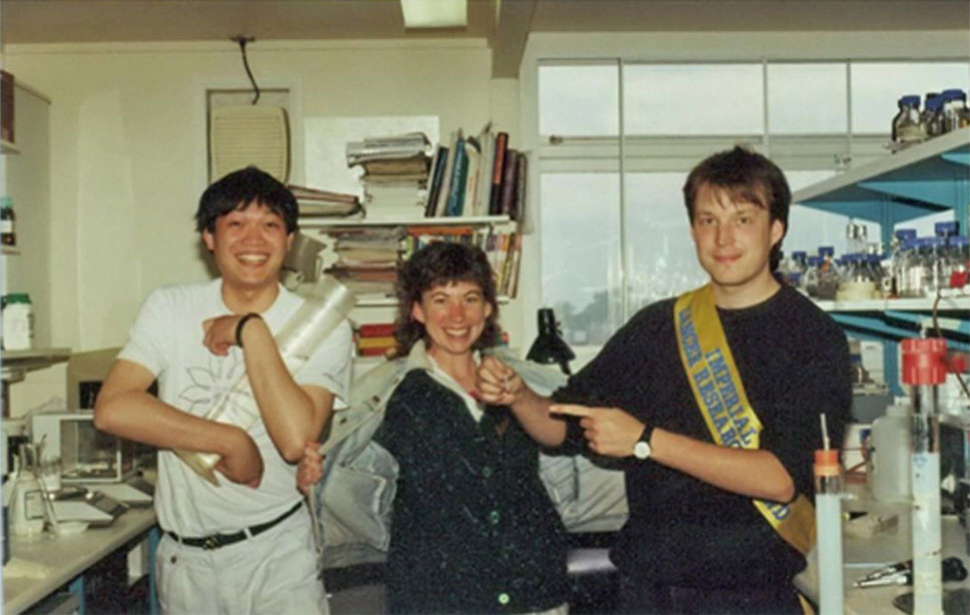
Dr. Jian Zhou (left) at the Tumor Virus Laboratory of Cambridge University

In 1977, Dr. Zhou was admitted to study at the Department of Medicine at Wenzhou Medical University where he obtained his Bachelor of Medicine degree. After graduation, he went on to study at the Zhejiang Medical University and obtained a Master’s degree in Medical Sciences. It was at the Zhejiang Medical University that Dr. Zhou began to study the human papillomavirus and developed his interest in the field of human pathology. In 1985, Dr. Zhou began his PhD studies in human pathology at the Henan Medical University where he continued his research in human papillomavirus. During that time, he also attended the Institute of Virology, Chinese Academy of Preventive Medicine and studied molecular virology and molecular cloning technology. During his Master’s degree and PhD studies, Dr. Zhou discovered that the development of esophageal cancer is associated with human papillomavirus infections. He received a national award in China for this discovery. In 1988, after he had obtained his PhD degree, Dr. Zhou undertook his first postdoctoral study at Beijing Medical University. During this period, he used vaccinia virus as a vector to express a specific protein *in vitro*, thereby laying the technological foundation for his later invention. In the same year, he was invited to work at the Tumor Virus Laboratory, in the Department of Pathology of Cambridge University to continue molecular biology research in the human papillomavirus under the guidance of Professor Lionel Crawford (Fig. 3). Dr. Zhou’s hard work, diligence and innovation allowed him to develop a clear understanding of the state of the research and the developmental direction for human papillomavirus research. He was soon publishing influential papers in journals such as the Journal of General Virology, Virology, Journal of Virology and other well-known international journals.

Dr. Zhou first met Professor Frazer, an immunologist from the University of Queensland, at Cambridge. Impressed by Dr. Zhou’s creative research ideas and bonded by mutual respect, Professor Frazer convinced Dr. Zhou to join him in Australia to pursue their common interest in human papillomavirus research. In 1990, Dr. Zhou joined Professor Frazer at the University of Queensland, Australia, starting the most important chapter of Dr. Zhou’s research career.

After multiple experiments, Dr. Zhou successfully used vaccinia virus as a carrier to express papillomavirus L1 and L2 capsid proteins in mammalian cells *in vitro* using recombinant DNA technology. The expressed capsid protein can be self-assembled into virus-like particles. In the same year, Dr. Zhou and Professor Frazer published these results in the Virology Journal (Zhou et al., 1991a). In June 1991, the University of Queensland applied for a provisional patent for the invention. In July of that year, Dr. Zhou and Professor Frazer reported their results at the International Conference of Human Papillomavirus in Seattle in the United States. This achievement was considered a major breakthrough in human medical history. Australian medical company, CSL and later Merck company, USA conducted large-scale animal and human clinical trials which ultimately verified the effect of human papillomavirus-like particles on the prevention of cervical cancer.

Dr. Zhou was an incredibly talented scientist. His contributions to papillomavirus and cancer biology research were exceptional. During his first four years at the Centre for Immunology and Cancer Research (later renamed The Diamantina Institute for Cancer, Immunology and Metabolic Medicine) at the University of Queensland, he published 11 first-author scientific papers in leading virology journals. This is an incredible achievement for any scientist involved in biomedical research. During his research career, Dr. Zhou made a number of world-renowned inventions and obtained twelve invention patents within eight years. One of these involved the discovery that the minor papillomavirus capsid protein L2 plays a crucial role in DNA binding and capsidification, revealing the importance of this capsid protein in the HPV life cycle (Zhou et al., 1991b). Dr. Zhou also found that L1 protein C-terminus does not affect the formation of capsids. This discovery led to the use of a chimeric capsid consisting of the C-terminal truncated L1 protein, and other T-cell epitopes of the early proteins of the virus, to produce both prophylactic and therapeutic vaccines. Another very novel study was genetic code (gene codon) optimization. In this study, he found that the HPV L1 and L2 capsid genes could not be expressed in most mammalian cells, but it can be expressed in yeast because the transfer ribonucleic acid (tRNA) in mammalian cells limits the expression of the capsid genes (Zhou et al., 1999). Dr. Zhou discovered that a capsid gene using the mammalian rare codon effectively expresses capsid protein in the infected terminal differentiation epithelium. Codon optimization and change could be used to improve significantly the efficiency of protein expression and improve the immunogenicity of the vaccine and opened up a new gene therapy technology (Zhou et al., 1999).

**Figure 4:**
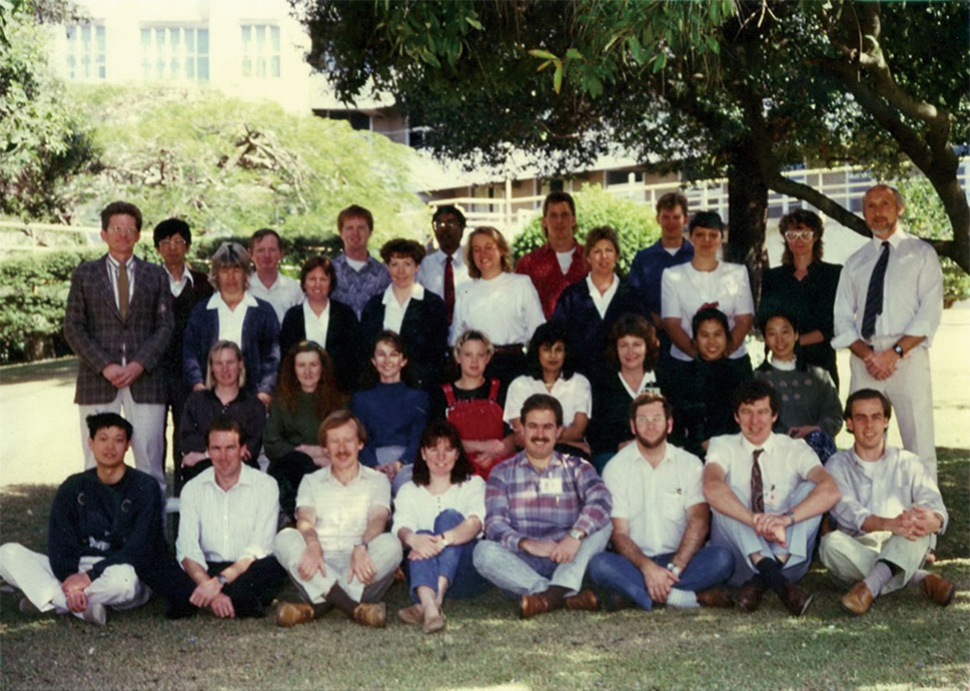
Dr. Jian Zhou (1st from left in the front row) at the Center for Cancer and Immunology Research of The University of Queensland

Dr. Zhou received his MD from the University of Queensland in 1994. After this he took on an associate professorship at the Loyola University School of Medicine in Chicago. He set up his own research group there and supervised PhD students and postdoctoral fellows working on different research projects in medical sciences. In 1996, Dr. Zhou returned to the University of Queensland and was appointed the principle Lions Research fellow and the Head of the Papillomavirus Structural Protein Laboratory at the Center for Cancer and Immunology Research (Fig. 4). In 1998, Dr. Zhou was awarded with three research grants by the Australian National Medical and Health Research Council, a grant by the American National Institute of Health, a grant by the American Cancer Foundation, two grants by the Cancer Council Queensland, and received funding for several other research projects from other resources as well as royalties from his patents. In that year, Dr. Zhou was the well-funded researcher receiving the largest amount of research funding in the history of the University of Queensland.

**Figure 5:**
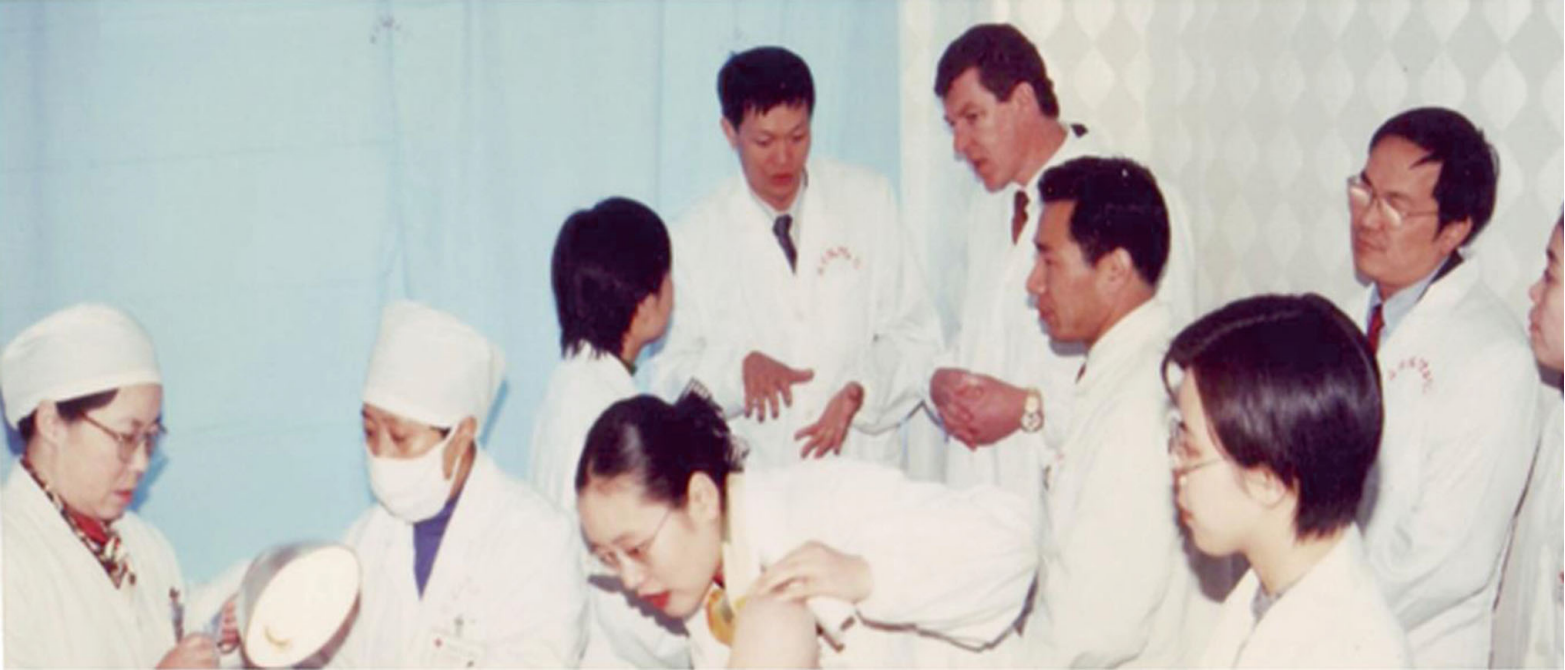
Dr. Jian Zhou (5th from the left) and Professor Ian Frazer (5th from right) at the first affiliation hospital of Wenzhou Medical University

Dr. Zhou was always proud of being a graduate of the Wenzhou Medical University and was always concerned with the development of teaching and scientific research at his alma mater. He travelled to Wenzhou Medical University to give lectures and guide scientific research and was selfless in spending his personal time and money inviting scholars from his alma mater to further their studies overseas (Fig. 5). One such example occurred in 1996 when Dr. Zhou was based in Chicago. When he found out that a scholar from his alma mater was studying in Boston, he immediately sent that person return flight tickets between Boston and Chicago so that they could get together to discuss how to support and carry out scientific research at the Wenzhou Medical University. Later, after Dr. Zhou joined the University of Queensland, he established a joint program between the Wenzhou Medical University and the University of Queensland to further scientific research collaboration and facilitate the exchange of medical students to accelerate the development of his alma mater. His family established the “Dr. Jian Zhou Foundation” to provide scholarships to medical students from Wenzhou Medical University, Dr. Zhou’s alma mater, to undertake PhD studies outside of China to train as medical research scientists. Dr. Zhou made a significant contribution to the development of Wenzhou Medical University as a well-known university in China and the world now.

**Figure 6:**
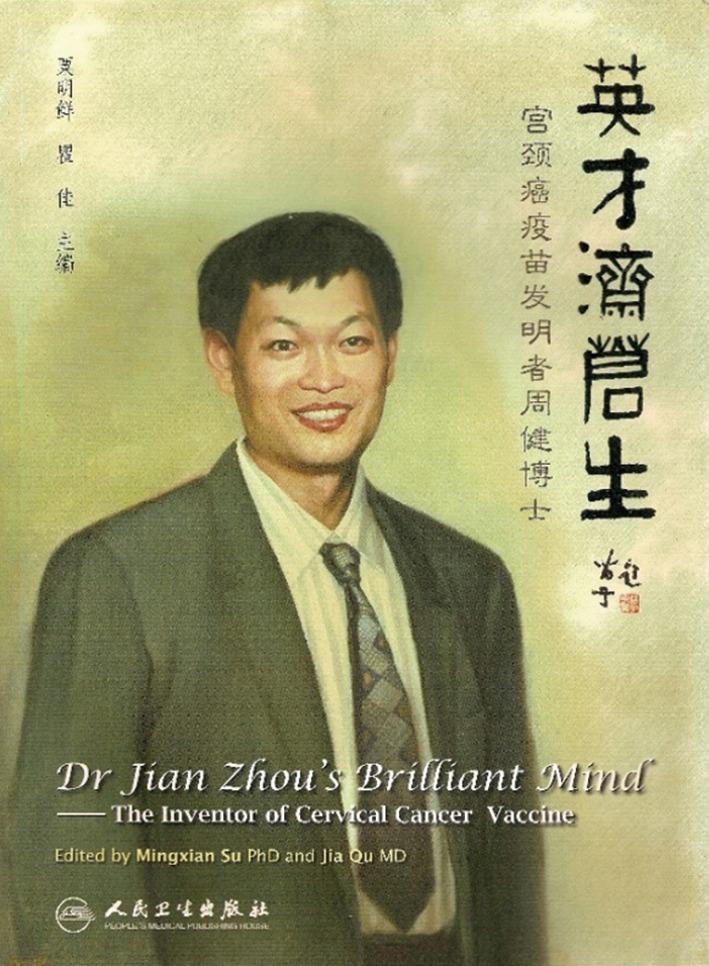
Cover photo of Dr. Jian Zhou memorial book

Dr. Zhou’s outstanding contributions to science and humanity have been recognised and commemorated. The Diamantina Institute for Cancer, Immunology and Metabolic Medicine of the University of Queensland established the annual Jian Zhou Memorial Oration at which world renowned scientists give lectures to commemorate Dr. Zhou. The Diamantina Institute has also named one of its conference rooms the Jian Zhou room. Since 2000, the annual international HPV Conference has held a number of memorials for Dr. Zhou. The Asian-Oceania Research Organization on Genital Infection and Neoplasia (AOGIN) in 2006 established the AOGIN Dr. Jian Zhou award as one of the three awards for best oral presentation. In 2006, the Queensland Government established the “Dr. Jian Zhou Smart State Fellowships Program”. The Queensland Government, the Australian Chinese Society and Australia-China Friendship Society organized the “Dr. Zhou Jian Memorial” and published a book entitled “Dr. Jian Zhou’s Brilliant Mind — The Inventor of Cervical Cancer Vaccine”《英才济苍生》(Fig. 6). Lastly, the vaccine against human papillomavirus (HPV) jointly invented by Dr. Zhou and Professor Frazer won the European Inventor Patent award in 2015.
